# Observe natural selection by evolutionary experiments in crops

**DOI:** 10.1007/s42994-025-00215-6

**Published:** 2025-05-20

**Authors:** Tian Wu, Shifeng Cheng

**Affiliations:** Agricultural Genomics Institute at Shenzhen, Chinese Academy of Agricultural Sciences, Shenzhen, 518124 China

**Keywords:** Evolutionary experiment, Barley, Diversity, Natural selection, Local adaptation

## Abstract

Evolutionary experiments provide a unique lens through which to observe the impacts of natural selection on crop evolution, domestication, and adaptation through empirical evidence. Enabled by modern technologies—such as the development of large-scale, structured evolving populations, high-throughput phenotyping, and genomics-driven genetics studies—the transition from theoretical evolutionary biology to practical application is now possible for staple crops. The century-long Barley Composite Cross II (CCII) competition experiment has offered invaluable insights into understanding the genomic and phenotypic basis of natural and artificial selection driven by environmental adaptation during crop evolution and domestication. These experiments enable scientists to measure evolutionary dynamics, in real time, of genetic diversity, adaptation of fitness-associated traits, and the trade-offs inherent in selective processes. Beyond advancing our understanding of evolutionary biology and agricultural practices, these studies provide critical insights into addressing global challenges, from ensuring food security to fostering resilience in human societies.

## Understanding natural selection

Charles Darwin’s pioneering work on the theory of natural selection provided the first framework for understanding the evolution and domestication of species (Mayr 1982; Numbers 1981). In his seminal book in 1859, *On the Origin of Species* (Darwin 1859), followed by several other works with detailed explanations (Darwin 1872, 1875, 1876, 1877), Darwin proposed that biological diversity arises from small, gradual, and inherited variations that enhance an individual’s ability and fitness to compete, survive, and reproduce in specific environments. He emphasized that this diversity is shaped by advantageous variations in response to ecological pressures and competition, underscoring the critical role of the environment in driving natural selection (Zohary et al. 2012). Darwin’s insights formed the foundation for evolutionary biology, illustrating the intricate relationship between environmental conditions and genetic architectures in shaping phenotypic changes (Gepts 2004).

Despite the transformative impact of Darwin’s theories, many of his ideas remained largely conceptual and hypothetical, awaiting empirical validation in a real-world scenario. His theory of genetic inheritance, pangenesis, for instance, was based on speculative reasoning and was ultimately proven incorrect (Darwin 1875; Mayr 1982). Around the same period (1856–1865), Gregor Mendel investigated how pairs of differentiating characters are inherited across successive generations, using pea (*Pisum sativum*) as the model system. Unlike Darwin, Mendel’s work was experimental rather than conceptual, establishing the foundational laws of genetic inheritance (Mendel 1866). Mendel proposed the existence of inherited ‘factors’ that govern traits and explained the source of diversity, which is essential for understanding natural selection (Cheng 2022; Fisher 1936; Hartl and Orel 1992). However, both Mendel and Darwin lacked knowledge of the molecular basis of genetic factors and their underlying mechanisms—a mystery that would remain unsolved until the twentieth century, with the discovery of the DNA double helix and the advent of molecular biology (Avery et al. 1944; Watson and Crick 1953). This lack of molecular insights restricted researchers of their time to phenotypic observations and inferential reasoning, making it challenging to observe and quantify the genomic basis of natural selection.

Today, enabled by modern technologies, we have unprecedented opportunities to understand the genomic and phenotypic basis of diversity for a given organism (Annapurna et al. 2024). However, many breakthroughs are made in controlled laboratory settings using a simple model system, which fails to fully capture the complexities of the natural environment. Translating the knowledge from the lab to the field—where crops must adapt from their wild origins to diverse agricultural farming environments—remains a significant challenge (Huang and Han 2014). In addition, the rapid loss of genetic and phenotypic diversity as plants transition from the wild to the field and, eventually, to a simplified laboratory model can obscure crucial insights into how plants adapt to new environments.

## Evolutionary experiment as a powerful tool

Evolutionary experiments have proven to be powerful tools for studying the genomic and phenotypic basis of selection and adaptation, bridging the research gaps and integrating observations across the ‘Lab-Field-Wild’ continuum. By setting a series of specific parameters and environmental conditions, these carefully designed experiments connect fundamental research with real-world scenarios across time and space (Siepielski et al. 2009), with successful examples across unicellular organisms and many model systems (Barrick and Lenski 2009; Blount et al. 2008). A notable example is the long-term evolutionary experiment with *Escherichia coli*, pioneered by Richard Lenski (Cooper and Lenski 2000; Elena and Lenski 2003), which has provided invaluable real-time observations of evolutionary dynamics. Since 1988, this experiment has tracked genetic changes across over 70,000 bacterial generations, yielding insights into mutation rates, genetic stability, and the development of new metabolic capabilities (Blount et al. 2008). Similarly, evolutionary experiments with *Saccharomyces cerevisiae* (yeast) have advanced our understanding of adaptive mutations that confer fitness in specific environments and the rise of novel genetic networks and robustness (Holt 2009; Johnson et al. 2021). In *Drosophila* (fruit fly), experiments have further illustrated the role of sexual selection and genetic drift in real-world scenarios (Prout 1999). More recently, synthetic ecosystems have been created, allowing researchers to engineer environments that mimic certain natural or theoretical conditions to study evolutionary dynamics (Castle et al. 2024).

In higher plants, long-term experiments with *Arabidopsis thaliana* have facilitated the study of molecular mechanisms underlying genetic adaptation and trait evolution (Kronholm 2012), including traits such as flowering time and stress responses. Research on polyploidy in duckweed has shed light on how genome doubling affects plant adaptation, enhances phenotypic diversity, and influences ecological interactions (Bafort et al. 2023; Van de Peer et al. 2021; Wu et al. 2024). Polyploid plants often exhibit larger organs, such as bigger leaves and flowers, and increased biomass, which can contribute to evolutionary success and have practical implications for agriculture and conservation (Levin 1983).

The origin, spread, and domestication of modern major crops represents a natural ‘evolutionary experiment’, making research in this area crucial for advancing breeding practices (Endler 1986; Purugganan and Fuller 2009). The rapid expansion and global dissemination of crops have subjected them to strong selective pressures, including both artificial selections, for desired traits, and natural selection to diverse environments. However, a mechanistic understanding of how major crops transitioned from their centers of origin to adapt to new and varied environments remains largely elusive in the study of crop evolution and domestication. Long generation times and the slow pace at which genetic changes accumulate across generations in major crops complicate efforts to directly observe the molecular basis of natural selection and environmental adaptation over time (Savolainen et al. 2013; Stapley et al. 2010).

With the advent of omics technologies in the early twenty-first century, identifying the genomic loci underlying local adaptation in staple crops within specific environmental contexts is feasible (Tian et al. 2024; Chen et al. 2023). This task can be addressed through the ‘pattern-process-mechanism’ framework in evolutionary experiments (Hufford et al. 2013; Meyer et al. 2012): investigating the pattern (what), process (how), and mechanism (why) of natural selection and environmental adaptation. The ‘pattern’ refers to identifying what variations in genetic and phenotypic diversity across populations occur over time, such as shifts in allele frequencies. The ‘process’ involves understanding how the underlying forces, dynamic changes, and interactions that drive the rise of these patterns. The ‘mechanism’ seeks to explain why these evolutionary changes occur, focusing on the underlying genetic, molecular, ecological, or physiological factors.

## The Barley Composite Cross II (CCII) competition experiment

**Fig. 1:**
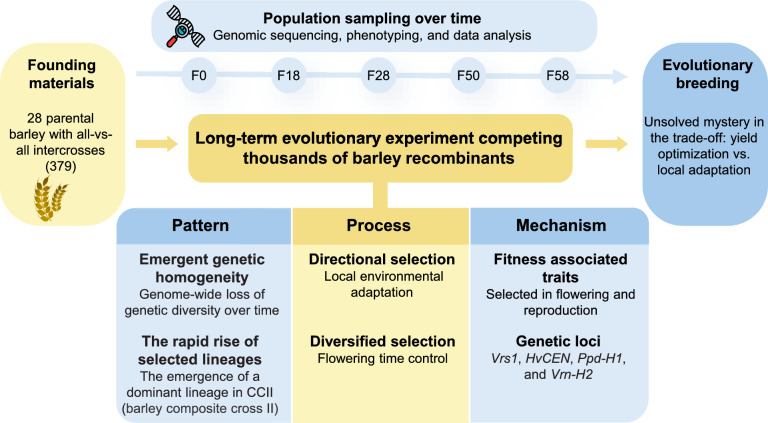
A brief summary of the major discoveries from the CCII evolutionary experiment by Landis et al. (2024)

The barley Composite Cross II (CCII) competition experiment, initiated in 1929, is one of the world’s oldest biological experiments and provides valuable insights into how barley adapts to changing environments and selective pressures (Landis et al. 2024). Barley was domesticated over 10,000 years ago in the Fertile Crescent (Morrell and Clegg 2007), followed by global dispersal, and has become an important source of nutrition for humans and livestock (Zohary et al. 2012). The CCII experiment offers a unique opportunity to observe and quantify real-time evolutionary dynamics in a self-fertilizing, annual crop, enabling the study of genetic variations and phenotypic changes across many generations. Through the CCII experiment (Fig. 1), researchers have tracked evolutionary changes (pattern), observing how allele frequencies shifted rapidly by natural selection (process) in response to local environmental conditions in Davis, California. By analyzing the founding population and subsequent generations (F18, F28, and F58), key genetic loci such as *Vrs1*, *HvCEN*, and *Ppd-H1*, which are associated with reproductive development, were identified as hotspots of selection (Landis et al. 2024). These loci were favored under local environmental pressures, demonstrating how specific genetic traits enhance survival and reproduction in particular climates (mechanism).

Contrary to Darwin’s view that evolution occurs in a slow and gradual manner (Charles 1859), the CCII experiment has revealed the rapid loss and homogenization of genetic diversity within just a few generations. Remarkably, this process led to the emergence of a single dominant lineage derived from North African parents that grew in Mediterranean climates like that of Davis, where the experiment was conducted. Meanwhile, the selected advantageous genetic loci conferring local environmental adaptation became nearly fixed in a matter of decades—without any conscious human-mediated selection. This finding suggests that adaptive alleles, which emerged during the early stages of agriculture, could dominate an ecogeographical location within just a few generations, challenging the prevailing assumption that high initial genetic diversity within a population will sustain long-term variability (Meyer and Purugganan 2013; Purugganan and Fuller 2009). Natural selection driving genetic homogeneity rapidly provides insights for understanding the potential vulnerabilities of crop species that may arise from reduced genetic diversity, impacting their ability to adapt to future environmental changes (Purugganan and Fuller 2009).

Another interesting discovery was the dynamic interplay between directional and stabilizing selection in the CCII population. Directional selection initially favored certain alleles that conferred advantages in the local environment, driving rapid changes in allele frequencies. However, as these advantageous traits became widespread, stabilizing selection began to play a crucial role. For example, the CCII experiment revealed the genetic basis of fitness-associated traits, particularly the flowering time traits, which are critical for reproductive success. These traits were subject to stabilizing selection, driving the population toward intermediate phenotypes by gradually removing the earliest flowering genotypes. The finely tuned balance between adaptation and stabilization demonstrated a two-step selection process, in which an initial rapid adaptation is followed by a phase of stabilization, ensuring that the population is well suited to its local environment. Such insights would be difficult to capture or could be misinterpreted without the holistic perspective provided by long-term evolutionary experiments. This highlights an underappreciated mechanism of selection in maintaining genetic diversity and ensuring the long-term adaptability of crop populations.

Similarly, Rudman et al. (2022) also reported a study that provided direct evidence of adaptive tracking, where *Drosophila melanogaster* populations continuously adapted to rapidly changing environmental conditions over short time scales. This was demonstrated by the rapid and repeated phenotypic and genomic changes observed across multiple traits and loci within just a few generations. This rapid evolution challenges the traditional view that adaptation is a slow process relative to environmental change. The study also highlighted the role of fluctuating selection, where the direction and strength of selection changed over time, leading to reversals in the adaptive trajectory of certain traits. This demonstrated that adaptation was not a linear process but rather a dynamic one, with populations continuously tracking environmental changes. This study illustrated that adaptation involved multiple loci across the genome, with tens to hundreds of genetic variants under selection. This polygenic nature of adaptation suggests that even complex traits can evolve rapidly in response to environmental pressures.

The trade-off between local adaptation and high-yield traits observed in the CCII experiment highlights a key challenge in crop breeding: while local adaptation enhances survival and competitiveness, under specific environmental conditions, it often comes at the cost of maximizing yield. This reflects a resource allocation dilemma, where traits that confer fitness in natural settings may divert resources away from productivity (Simmonds 1991). The evolutionary breeding approach, resembling early domestication processes, successfully promoted local adaptation but lagged behind pedigree-based methods in achieving rapid yield improvements. Understanding the genetic and physiological mechanisms behind this trade-off is crucial for developing strategies that balance resilience, diversity, and yield, ensuring sustainable crop improvement in changing environments.

## Natural diversity and environmental adaptation in other crops

Although most crops have not been subjected to evolutionary experiments or such experiments are challenging to conduct, the century-long CCII evolutionary experiment with barley has offered valuable insights and serves as a model for designing similar studies in other crops. For instance, the Illinois Corn Experiment (Harlan and de Wet 1971), initiated in 1896, is one of the longest-running plant breeding studies, which focuses on selecting maize for high and low oil and protein content over generations.

A study on sunflowers (Baute et al. 2015) demonstrates how structural variants in large haplotype blocks can quickly drive diversity loss and local adaptation. These authors identified haplotype blocks that are regions of the genome with limited recombination, caused by genomic inversion events. These blocks, where natural selection was favored, are strongly associated with traits that are critical for local environmental adaptation, such as flowering time. This study also emphasizes the importance of observing evolutionary dynamics in real-world scenarios, such as how crops and wild plants adapt to specific environmental pressures, how adaptive haplotypes, gene flow by introgression, and fitness-associated traits confer ecotypic differentiation and even speciation.

Many similar studies have been conducted in the field of population and evolutionary genetics in crops. For instance, the geographical adaptation of rice to soil nitrogen content was investigated by growing a core rice diversity panel across different locations (Liu et al. 2021). This study identified the genomic and phenotypic basis of this adaptation and reported that the *OsTCP19-H* allele, beneficial in nitrogen-deficient environments, has been largely lost in modern cultivars, due to agricultural practices like the widespread use of nitrogen fertilizers. This highlights the significant role of natural selection in shaping the adaptation of rice varieties to local soil nitrogen conditions during domestication. It also underscores how specific alleles confer advantages in response to environmental pressures. This work emphasizes the need to maintain and reintroduce adaptive alleles, such as *OsTCP19-H*, and mirrors findings from the CCII experiment, where preserving genetic diversity is key to enabling crops to adapt to changing environmental conditions.

In wheat, a study on the century-old *A.E. Watkins* landrace collection of bread wheat revealed that it contains a vast reservoir of previously untapped genetic diversity (Cheng et al. 2024). Modern wheat cultivars are derived primarily from only two of the seven ancestral groups (AGs) identified in the Watkins collection, leaving five AGs with unique genetic variants that are largely absent in contemporary wheat. These untapped AGs hold valuable adaptive alleles and haplotypes that could be critical for future trait breeding efforts, particularly in enhancing traits such as yield, stress resilience, and disease resistance in the face of climate fluctuations. This study also underscores the importance of genetic diversity as a critical resource for crop adaptation and resilience.

## Future prospects

Long-term evolutionary experiments are invaluable for examining in detail the pattern, process, and mechanism of molecular diversity driven by both artificial and natural selection. These experiments offer direct evidence of how crops adapt to their environments (Weigel and Nordborg 2015), tracing the historical spread and domestication of crops while providing predictive insights into designing climate-resilient crops for future environmental changes. These experiments not only hold significant agronomic value in a rapidly changing world, enabling systematic studies that unlock the genetic architecture driving adaptive evolution in plants (Foley et al. 2011; Larson and Fuller 2014), but also have great implications for sustainable development in our human societies and beyond.

The integration of advanced technologies offers unparalleled opportunities to unravel the genetic and ecological mechanisms that drive adaptation, trait evolution, and agricultural sustainability (Lucas et al. 2024). One of the most promising methodologies in evolutionary genetics today is the “Evolve by Re-sequencing” strategy, which combines real-time genomic sequencing with controlled evolutionary experiments (Long et al. 2015). By monitoring genetic changes in populations, as they evolve in response to selective pressures, researchers can directly link genetic variation to phenotypic outcomes. Studying how crops such as wheat, barley, and rice evolve under different environmental stresses, scientists can identify alleles that are beneficial under specific conditions, such as high-temperature tolerance, salt resistance, and increased nutrient use efficiency.

This information can be directly applied to breeding programs, helping to develop crop varieties that are better suited to the challenges of the future. For example, the study of crops under long-term experimental conditions, such as the Composite Cross II (CCII) barley experiment, has already revealed valuable insights into the evolution of agronomically relevant traits. On the other hand, by studying how wild relatives of crops evolve in response to natural and anthropogenic factors, researchers can better understand the processes that have shaped the genetic makeup of modern crop species and leverage this knowledge to guide future breeding strategies.

Furthermore, careful designs of evolutionary experiments, with a series of controlled environmental settings, to study complex traits, such as C4 photosynthesis (Swift et al. 2024) or legume root nodule symbiosis (Griesmann et al. 2018), could provide insights into how evolutionary novelties arose and diversified under specific ecological conditions. By simulating selective environments and tracking the genetic and physiological changes that accompany the evolution of these complex traits, researchers can measure the different aspects and dimensions of diversity derived from the novel traits and how these can be incorporated into modern crops. Although these experiments will be long term, with some challenges to overcome, they could lead to a mechanistic understanding of the high-performance natural traits, thereby leading to the development of crops with enhanced photosynthetic efficiency, better nitrogen fixation, and increased resilience to drought and nutrient limitations, ultimately improving the sustainability and productivity of agricultural systems.

As the genomics revolution is leading to a genetics revolution and, further, to a breeding revolution, the integration of high-throughput sequencing, big data analysis, and artificial intelligence (AI) will be essential in transforming evolutionary experiments into practical solutions for agriculture. AI and machine learning algorithms can help researchers analyze vast structured datasets generated in time and space. In the future, the combination of genomic data with environmental variables could enable scientists to model evolutionary trajectories and predict how crops will adapt to future climates. This would allow for the development of predictive breeding strategies, where crops are engineered not only for current environmental conditions but also for future scenarios that may include extreme weather events, changing disease dynamics, and shifting soil properties.

## Conclusions

Evolutionary experiments stand at the intersection of foundational biological principles and cutting-edge technologies, offering transformative insights into plant science, crop breeding, and agriculture. The legacy of pioneers such as Charles Darwin, who first articulated the theory of natural selection; Gregor Mendel, whose discoveries laid the foundation for genetic inheritance; and James Watson and Francis Crick, who elucidated the structure of DNA, has shaped our understanding of evolution and natural selection. Their work highlights the enduring power of investigating the genomic and phenotypic foundations of evolutionary processes. By bridging the gap between fundamental research and real-world challenges, evolutionary experiments provide unparalleled opportunities to decipher the genetic basis of adaptive traits and help develop resilient, high-yielding crops that balance the demands of environmental adaptation with global food security needs in a sustainable manner. These experiments offer invaluable insights and inspiration for addressing the pressing challenges we face in today’s world.

## Data Availability

Data sharing is not applicable to this article as no datasets were generated or analyzed during the current study.
